# Outcomes of a postoperative perfluorocarbon liquid tamponade for complex retinal detachments: 12 years of experience in southern Thailand

**DOI:** 10.1186/s12886-020-01600-z

**Published:** 2020-09-01

**Authors:** Patama Bhurayanontachai, Usanee Seepongphun

**Affiliations:** Department of Ophthalmology, Faculty of Medicine, Prince of Songkla University, 15 Karnjanawanich Road, Hat Yai, Songkhla, 90110 Thailand

**Keywords:** Giant tear, Perfluorocarbon liquid, Retinal detachment, Tamponade, Vitrectomy

## Abstract

**Background:**

The study evaluates both functional and anatomical outcomes of retinal detachment (RD) repair by vitrectomy and perfluorocarbon liquid (PFCL) tamponade.

**Methods:**

A retrospective chart review of patients who underwent vitrectomy using PFCL tamponade for RD repair from causes such as giant tear, chronic RD, or RD with previously failed surgery.

**Results:**

This study included 122 eyes from 121 patients. One-hundred fourteen eyes (93.5%) had baseline vision worse than 20/200. The median duration of intraocular PFCL retainment was 14 days before gas or silicone oil replacement. The retinal reattachment rate was 80.3%. At 1 year, the retention probability of retinal reattachment was 0.84 (95% confidence interval, 0.77–0.91). Although visual improvement was found in 45.9% of patients, the median of final vision was not different between baseline and the last visit.

**Conclusion:**

The rate of retinal reattachment operated with a short- to medium-term PFCL tamponade achieved a high satisfaction rate. However, postoperative hypotony was a predictor for unfavorable visual and anatomical outcomes.

## Background

Perfluorocarbon liquid (PFCL) is a synthetic, fluorinated, carbon-containing compound. It is clear, colorless, odorless, and heavier than water. The PFCL does not affect the intraoperative view. The physical properties of PFCL make it an ideal tool for flattening the retina during vitreoretinal surgery for many types of pathologies, such as retinal detachment (RD) with severe proliferative vitreoretinopathy (PVR) [1–4], giant retinal tear [5–10], and pediatric RD [11]. Patients with chronic RD or those who underwent previously failed surgery are frequently accompanied by severe PVR, which sometimes produces severe peripheral retinal contraction and shortening and may require a retinectomy. During injection, the high specificity gravity of PFCL allows the detached retina to be smoothly flattened from the posterior pole and displaces unwanted subretinal fluid anteriorly passing through the presenting retinal breaks or peripheral retinectomy. In contrast, an air-fluid exchange flattens the retina from the periphery and pushes subretinal fluid toward the posterior pole; therefore, a retinotomy is required. Using PFCL can also be an alternative or adjunctive to the standard scleral buckling procedure.

A large multicenter study reported results of intraoperative PFCL-assisted vitrectomy for complex RD with PVR [2]. Other case series have reported results of primary vitrectomy with a short-term postoperative PFCL tamponade [3–8]. A few studies have reported results of reoperation or rescued vitrectomy with a medium- and long-term PFCL tamponade in complex RD [12–15]. However, studies of a short- to medium-term PFCL use in an Asian population are only few. In the literature, complications after PFCL tamponade included subretinal PFCL, PFCL-related intraocular inflammation, elevated intraocular pressure (IOP), and epiretinal membrane (ERM) [8, 12, 13, 15].

We conducted a retrospective study of patients who underwent vitreoretinal surgeries with a short- to medium-term PFCL tamponade. This study primarily aims to demonstrate both anatomical and functional results after PFCL placement and removal. The secondary aim was to evaluate factors that affect the outcomes.

## Methods

### Data collection

This retrospective study was approved by the Ethics Committee of the Faculty of Medicine, Prince of Songkla University, Songkhla, Thailand (REC number 58–364–02-3). The study included patients with RD who underwent pars plana vitrectomy (PPV) using postoperative PFCL tamponade (with or without scleral buckling procedure) in the tertiary referral university hospital. Data collection was performed using the hospital’s electronic database to identify patients who had RD and were treated by standard three-port PPV. The search terms used were the following International Statistical Classification of Diseases-10 and International Statistical Classification of Diseases-9-Clinical Modification codes: (1) H33.0 RD with retinal break, (2) H33.4 Traction detachment of retina, (3) H33.5 Other RDs, and (4) 14.74 Other mechanical vitrectomy by posterior approach.

Eligible patients were determined by manual search. The inclusion criteria included rhegmatogenous RD (RRD) from any cause, tractional RD (TRD), and any RRD or TRD having a previously failed treatment. The exclusion criteria were any concomitant ocular diseases, such as macular choroidal neovascularization, dense cataract, advanced or uncontrolled ocular disease (e.g., glaucoma, uveitis, uncontrolled systemic disease), pregnancy, or loss of critical data records.

The patients received 20- or 23-gauged PPV with PFCL (Perfluoron; Alcon Laboratories, Inc., Fort Worth, TX) tamponade by any of three vitreoretinal surgeons in our unit. The surgical techniques of all surgeons were performed in a similar way. A core vitrectomy was done, followed by posterior vitreous detachment induction and peripheral vitreous trimming. As much PVR as possible was removed before flattening the retina by PFCL. All patients were instructed to limit activities and lie in bed in a supine position during the postoperative period in the ward. Patients were allowed to ambulate in upright position only for meals and toileting. After 1 to 2 weeks, either perfluoropropane gas (Ispan C_3_F_8_; Alcon Laboratories, Inc.) or silicone oil (Oxane 5700; Bausch and Lomb, Inc., Rochester, NY) tamponade was chosen for replacing PFCL in the second operation. Patients who required additional RD repair by reinfusion of PFCL were also excluded from the study. Any further operations to fix the residual RD, as well as cataract surgery, to achieve patient’s maximal visual potential were allowed.

### Data management

All eyes underwent at least two operations: PFCL placement and PFCL removal. The date of surgery with PFCL placement was defined as the starting date of the study. Data were recorded at 1 week, 1 month, 3 months, and 6 months after PFCL removal, as well as at the last visit. These included best-corrected visual acuity (BCVA), postoperative retinal status, and postoperative complications.

The primary outcome was reattachment of the retina (anatomical outcome). The secondary outcomes were vision improvement (functional outcome) and complications during the follow-up period. Vision improvement was determined by an increase of at least one line of BCVA by Early Treatment Diabetic Retinopathy Study chart or a one-step gain of nonnumerical vision test (e.g., hand motion improved to counting of fingers) at the last visit compared with the baseline BCVA. The final visual outcome was then classified into two groups: “better or improved VA” and “stable or worsened VA”. A logarithm of the minimum angle of resolution (logMAR) VA was also used for vision calculation and comparison. All defined complications were elevated IOP greater than 25 mmHg, hypotony (IOP, < 5 mmHg), cataract progression, optic disc atrophy, ERM, corneal decompensation or edema, and retained intraocular PFCL bubble.

### Statistical analysis

Baseline characteristics and important variables were analyzed in an R program version 3.4.0 (The R Group, Vienna, Austria) with Epicalc software (Brixton Health, www.brixtonhealth.com). The continuous variables were described as mean ± standard deviation (SD) or as median with interquartile range (IQR), depending on data distribution. The relationships between the status of either reattachment or final VA change were evaluated by Student’s *t* test or the Wilcoxon rank-sum test for continuous data and by chi-square test or Fisher’s exact test for categorical data. The recurrence time of RD after PFCL removal was used to plot a Kaplan–Meier survival graph. To identify a correlation of variables to the final outcomes, significant variables in a univariate analysis were introduced into a multivariate analysis. A logistic regression was applied to predict factors on retinal reattachment and final visual outcome. A result was considered statistically significant when there was a *P* value less than 0.05.

## Results

### General characteristics

Table 1 summarizes the general patient characteristics. The raw data used to support the findings of this study are available from the corresponding author upon request. The study included 122 eyes from 121 patients. The BCVA ranged from 20/50 to perception of light. Most of the eyes (63.1%) began with hand motion. For 76 eyes in which RD was not chronic and the onset of visual symptoms could be obtained, the mean duration from the onset to the operation with PFCL placement was 125.8 ± 155 days. The mean interval between the latest vitrectomy and the starting date of the study was 173.6 ± 395.2 days (range, 3–2527 days). Four eyes with giant tear and five eyes with chronic RRD were myopic. Cytomegalovirus retinitis accounted for three cases of chronic RRD. Trauma cases included penetrating ocular injuries, traumatic RRD with vitreous hemorrhage, and intraocular foreign bodies. Previous vitreous surgeries in 42 eyes (34.4%) included simple PPV without vitreous substitution, PPV with gas or silicone oil tamponade, PPV for intraocular foreign body removal, and anterior vitrectomy.

**Table 1:** General characteristics of patients

Characteristics	***N*** = 122	(%)
Age (y)		
Mean	42 (±18.5)	
Median	43.5 (6–83)	
Sex		
Male	87	71.3
Female	35	28.7
Laterality		
Right	66	54.1
Left	56	45.9
Preoperative BCVA		
> 20/70	2	1.6
20/70–20/200	6	4.9
< 20/200	114	93.5
Preoperative logMAR BCVA		
Mean	1.8 (±0.4)	
Median	2 (0.4–2.3)	
Lens status		
Phakic	75	61.5
Pseudophakic	33	27.0
Aphakic	13	10.7
Unknown	1	0.8
Average time of symptom-to-PFCL placement (d)	125 (±156.1)	
Duration of PFCL placement (d)		
Mean	12.4 (±2.8)	
Median	14 (5–21)	
Cause: PFCL placement as a primary surgery (*n* = 80)		
RRD-chronic	32	26.2
RRD-giant tear	27	22.1
TRD	11	9.0
Trauma	11	9.0
PFCL placement as a rescue surgery (*n* = 42)		
RRD-failed repair	30	24.6
RRD-giant tear	5	4.1
TRD	2	1.6
Trauma	4	3.3
Reattachment:		
Yes	98	80.3
No	22	18.0
Cannot be evaluated	2	1.6
Interval before failed reattachment, *n* = 22 (wk)		
Mean	40.8 (±53.1)	
Median	30.2 (1.7–243.1)	
Follow-up time (wk)		
Mean	101.6 (±101.0)	
Median	67.6 (2.7–461.1)	
Final logMAR VA		
Mean	1.6 (±0.7)	
Median	2 (0.1–3.0)	

*BCVA* best-corrected visual acuity; *logMAR* logarithm of the minimum angle of resolution; *PFCL* perfluorocarbon liquid; *RRD* rhegmatogenous retinal detachment; *TRD* tractional retinal detachment; *VA* visual acuity

In 30 eyes with previously failed RD repair, 64.5% had received silicone oil endotamponade, and the rest had received gas endotamponade before PFCL placement. A retinectomy was performed in 36.6% of these eyes. A retinectomy with PFCL placement was also performed in 31 (53.1%) eyes with chronic RD and 13 (76.9%) eyes with severe TRD.

After the operation with PFCL placement, 97.5% of the retinas were reattached at the time of PFCL removal. When PFCL was removed, 101 eyes (82.8%) were replaced by silicone oil and 21 eyes (17.2%) were replaced by gas. Of the 80 eyes (65.6%), 38 had additional operation for silicone oil removal, 33 had combined surgery for cataract extraction and silicone oil removal, 4 required additional vitrectomy for residual RD repair, 3 had cataract extraction only, 1 had an ERM peeling, and 1 had partial silicone oil release to reduce elevated postoperative IOP.

Of the 11 eyes (9%), 3 had cataract surgery or secondary intraocular lens insertion, 3 had a recurrent detachment repair with silicone oil tamponade, 2 had combined surgery for cataract extraction and silicone oil removal, 1 had four operations for silicone oil removal, 1 had an intravitreal antibiotic injection for suspected endophthalmitis, and 1 had an evisceration for phthisis bulbus.

Three eyes (2.5%) had fifth operations, one each for silicone oil removal, intraocular lens reposition, and band keratopathy removal. Only one patient, who had undergone a band keratopathy removal, underwent a sixth operation for silicone oil removal. A residual PFCL droplet in the anterior chamber or in the vitreous cavity was noted in six eyes (4.9%). However, none of them were confronted with subretinal PFCL.

**Fig. 1:**
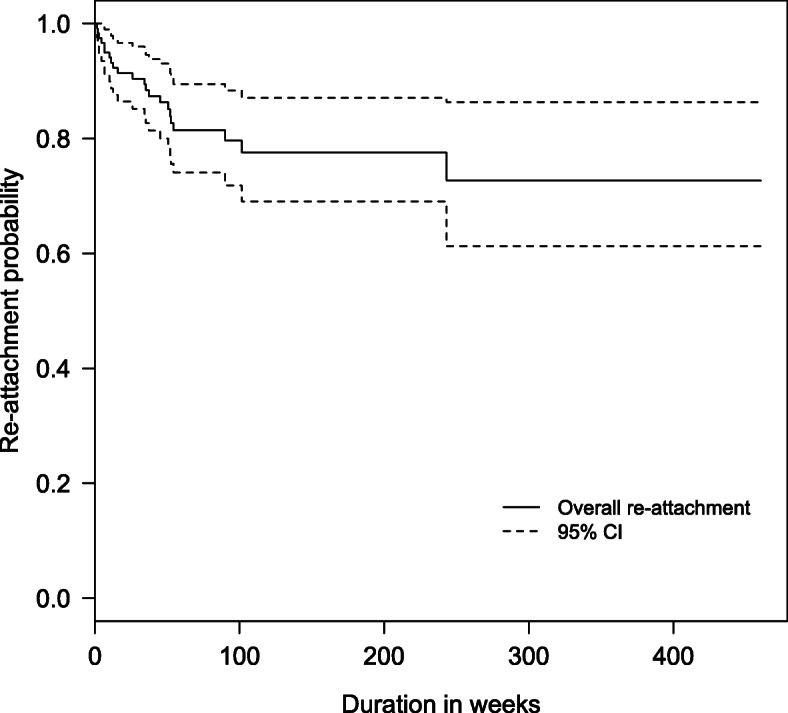
Kaplan-Meier estimation of retention of retinal re-attachment by time

Retinal reattachment was achieved in all cases during PFCL retention in the vitreous cavity. The reattachment rate at the last visit was 80.3%. Figure 1 demonstrates that the recurrent RD after PFCL removal was more likely to occur in the early phase. At 1 year, the probability of retention of retinal reattachment was 0.84 (95% confidence interval, 0.77–0.91). By the end of the eighth year, nearly three-quarters of patients who returned for follow-up still had their operated retinas attached.

Table 2 demonstrates groups of postoperative BCVA at different time points during the follow-up period. The vision of most eyes tended to improve over time. The final BCVA improved from baseline in 56 eyes (45.9%), remained stable in 46 eyes (37.7%), and worsened in 20 eyes (16.4%).

**Table 2:** Postoperative best-corrected visual acuity

Visual acuity at:	1 week	1 month	3 month	6 month	Last visit
Number of eyes (n)	119	120	115	110	122
> 20/70	1 (0.8%)	3 (2.5%)	5 (4.3%)	3 (2.7%)	12 (9.8%)
20/70–20/200	10 (8.4%)	23 (19.2%)	25 (21.7%)	22 (20.0%)	25 (20.5%)
< 20/200–PL	108 (90.8%)	94 (78.3%)	85 (73.9%)	85 (77.3%)	76 (62.3%)
No PL	0	0	0	0	9 (7.4%)

The most common complication was an immediate postoperative IOP elevation (51.2%), which usually occurred the next day after PFCL placement. IOP elevation and hypotony occurring after PFCL removal was found in 25.4 and 23% of eyes, respectively. Optic nerve atrophy and cataract progression were equally found in 22.1% of eyes. ERM occurred in 13.1% of eyes. A case of suspected late postoperative endophthalmitis, occurring at 5 years after the patient’s third operation of combined phacoemulsification and silicone oil removal, was successfully treated with a single injection of intravitreal antibiotics.

The univariate analyses of the effect of pre- and postoperative variables on retinal reattachment and visual outcome are demonstrated in Tables 3 and 4. Although male sex was predominant in the study, sex had no effect on either anatomical or visual outcome (*P* = 0.828 and 0.405, respectively).

**Table 3:** Relationship between preoperative factors, retinal reattachment, and the final VA status

	Reattachment		*P* value	Final VA Status		*P* value
	No (n = 22)	Yes (*n* = 98)		Better (*n* = 56)	Stable or worse (*n* = 66)	
Mean age (SD)	34.3 (±17.6)	43.8 (±18.1)	0.027*	41.9 (±16.8)	42.1 (±19.9)	0.953
Sex						
Female	6 (27.3)	29 (29.6)	1.000	14 (25)	21 (31.8)	0.529
Male	16 (72.7)	69 (70.4)		42 (75)	45 (68.2)	
Laterality						
Left eye	16 (72.7)	40 (40.8)	0.013*	24 (42.9)	32 (48.5)	0.660
Right eye	6 (27.3)	58 (59.2)		32 (57.1)	34 (51.5)	
Median logMAR VA (IQR)	2 (2–2)	2 (1.7–2)	0.569	2 (1.6–2)	2 (2–2)	0.134
Lens						
Aphakic	2 (9.1)	11 (11.3)	0.101	3 (5.5)	10 (15.2)	0.214
Pseudophakic	2 (9.1)	29 (29.9)		15 (27.3)	18 (27.3)	
Phakic	18 (81.8)	57 (58.8)		37 (67.3)	38 (57.6)	
Cause						
Chronic RRD	6 (27.3)	26 (26.5)	0.970	12 (21.4)	20 (30.3)	0.001*
Giant tear	6 (27.3)	26 (26.5)		24 (42.9)	8 (12.1)	
Trauma	3 (13.6)	12 (12.2)		5 (8.9)	10 (15.2)	
Previously failed RD repair	4 (18.2)	24 (24.5)		13 (23.2)	17 (25.8)	
TRD	3 (13.6)	10 (10.2)		2 (3.6)	11 (16.7)	
Previous vitrectomy						
No	15 (68.2)	65 (66.3)	1.000	39 (69.6)	41 (62.1)	0.496
Yes	7 (31.8)	33 (33.7)		17 (30.4)	25 (37.9)	
Median time of symptom-to-PFCL placement (IQR)	64 (20–117)	55 (17–165)	0.804	45 (15–128.5)	82.5 (35.2–258.8)	0.098
Median duration of PFCL placement (IQR)	13.5 (10.2–14)	14 (11–14)	0.817	12 (10–14)	14 (11–14)	0.221

*IQR* interquartile range; *logMAR* logarithm of the minimum angle of resolution; *PFCL* perfluorocarbon liquid; *RD*, retinal detachment; *RRD* rhegmatogenous retinal detachment; *SD* standard deviation; *TRD* tractional retinal detachment; *VA* visual acuity

**Table 4:** Relationship between immediate- and medium-term postoperative conditions, final retinal reattachment, and VA status

	Reattachment		*P* value	Final VA Status		*P* value
	No (n = 22)	Yes (n = 98)		Better (n = 56)	Stable or worse (n = 66)	
Elevated IOP during PFCL placement						
No	13 (59.1)	46 (46.9)	0.427	24 (42.9)	35 (53.8)	0.306
Yes	9 (40.9)	52 (53.1)		32 (57.1)	30 (46.2)	
Elevated IOP after PFCL removal						
No	17 (77.3)	72 (73.5)	0.921	39 (69.6)	52 (78.8)	0.343
Yes	5 (22.7)	26 (26.5)		17 (30.4)	14 (21.2)	
Hypotony						
No	12 (54.5)	81 (82.7)	0.009*	52 (92.9)	42 (63.6)	< 0.001*
Yes	10 (45.5)	17 (17.3)		4 (7.1)	24 (36.4)	
Optic disc atrophy						
No	18 (81.8)	75 (76.5)	0.779	50 (89.3)	45 (68.2)	0.01*
Yes	4 (18.2)	23 (23.5)		6 (10.7)	21 (31.8)	
Cataract progression						
No	17 (77.3)	76 (77.6)	1.000	44 (78.6)	51 (77.3)	1.000
Yes	5 (22.7)	22 (22.4)		12 (21.4)	15 (22.7)	
Corneal decompensation						
No	16 (72.7)	84 (85.7)	0.201	52 (92.9)	50 (75.8)	0.022*
Yes	6 (27.3)	14 (14.3)		4 (7.1)	16 (24.2)	
ERM						
No	20 (90.9)	84 (85.7)	0.733	48 (85.7)	58 (87.9)	0.933
Yes	2 (9.1)	14 (14.3)		8 (14.3)	8 (12.1)	

*ERM* epiretinal membrane; *IOP* intraocular pressure; *PFCL* perfluorocarbon liquid; *VA* visual acuity

Table 5 summarizes the results of multivariate analyses of characteristics regarding anatomical and functional successes. The significant predicting factors for retinal reattachment were right eye, lens status, and the absence of postoperative hypotony. Pseudophakic eyes appeared to have a better anatomical outcome than aphakic and phakic eyes. The significant predicting factors for better final BCVA were RD caused by giant tear and the absence of postoperative hypotony.

**Table 5:** Logistic regression of predicting factors on retinal reattachment and final visual outcome

Predicting reattachment			
	Adjusted OR (95% CI)	*P* value (Wald’s test)	*P* value (LR test)
Laterality			
Left eye	1	0.013*	0.009*
Right eye	4.2 (1.35–13.01)		
Lens status			
Aphakic	1	0.440	0.041*
Pseudophakic	2.53 (0.24–26.75)	0.274	
Phakic	0.38 (0.07–2.15)		
Hypotony			
Yes	1	0.005*	0.005*
No	5.18 (1.62–16.52)		
Predicting improved final BCVA			
Cause			
Trauma	1	0.858	0.004*
Chronic RRD	1.13 (0.29–4.38)	0.016*	
Giant tear RRD	5.66 (1.38–23.20)	0.495	
Previously failed RD repair	1.61 (0.41–6.31)	0.350	
TRD	0.40 (0.06–2.73)		
Hypotony			
Yes	1	0.002*	< 0.001*
No	6.83 (2.07–22.52)		

## Discussion

Our study included 122 eyes from 121 patients with varied retinal pathologies. Difficult cases such as chronic RRD with PVR and previously failed surgery for retinal attachment comprised more than half of the patients included. The duration from the patient’s symptoms to the starting date of this study was 125 ± 156.1 days. Therefore, the chronicity of the RD would be at least 4 or more months on average. The longer the duration of RD, the higher the risk of surgical failure in these patients. Therefore, silicone oil was a preferred choice of vitreous substitution to hold the retina in place after the retina was successfully reattached by PFCL (81.1%, oil; 17.2%, gas) and to lessen the risk of postoperative hypotony.

The result showed that a short- and medium-term PFCL retainment (≤20 days) was safe enough for complex RD repair in real-life practice. The success rate of reattachment was 80.3% of eyes. The causes of RD had an effect on the final visual outcome, but not on the retinal reattachment rate. A TRD, which was mostly caused by proliferative diabetic retinopathy, had the worst visual prognosis. The eyes with giant tear achieved better final vision than eyes with RD from other causes. This may be because of the fact that 84.4% of eyes with giant tear underwent a PFCL-assisted surgery on the first attempt and PVR was not involved much in these surgeries. The success of using PFCL in eyes with chronic RD and with reoperation was compromised from the preoperative PVR. An interval from the onset of symptoms to the time of PFCL placement did not affect the reattachment rate.

The majority of patients started with baseline VA worse than 20/200. The visual level at the last visit had improved in 45.9% of eyes. Eyes with giant tear accounted for the highest percentage of postoperative vision improvement. Mean logMAR VA increased from 1.8 ± 0.4 at baseline to 1.6 ± 0.7 at the last visit. However, the median logMAR VA was not different between baseline (2, 0.4–2.3) and last visit (2, 0.1–3.0). Other studies have reported a visual improvement that varied from 56.8 to 60% [1, 4].

The retinal reattachment rate with the use of PFCL in our study was comparable to the rate of 76–100% reported by other studies [2–13], even though postoperative visual improvement was not as good as the anatomical improvement.

Our results showed a high rate of early postoperative IOP elevation in the next day after PFCL placement, which could be controlled by medication in all patients. Other reports found a transient IOP elevation that varied from none to 35.9% at any time postoperatively [8, 13]. We also found that an IOP fluctuation was the main problem associated with either PFCL placement or PFCL removal. However, this did not affect the reattachment rate. Postoperative hypotony was noted to be a strong predictor for both anatomical and unfavorable visual outcomes.

Retinal toxicities and foreign body reaction from PFCL were occasionally reported [15, 16]. There was no sign of retinal toxicity from the PFCL reported in our study. However, optic nerve atrophy was found in 22.1% of the overall cases in the late postoperative period, and this compromised a visual recovery in successful cases. Either using PFCL or repeated surgeries could account for late optic nerve atrophy.

There were some limitations in this study. First, our center is a tertiary referral hospital; therefore, selection bias might have occurred. Second, the study was conducted retrospectively. Third, a small number of patients with TRD made it difficult to strongly predict the final visual prognosis. Finally, some patients still had their eyes filled with silicone oil at the end of the data collection period; therefore, the recorded BCVA might not reflect their true, final visual outcomes.

## Conclusion

Using short- to medium-term PFCL tamponade for complex RD was anatomically successful in 80.3% of patients in this study. At least one-line or one-level vision gain was achieved in 45.9% of eyes. Postoperative hypotony was a strong predictor for both anatomical and unfavorable visual outcomes.

## Data Availability

The datasets used and/or analysed in the current study are available from the corresponding author on reasonable request.

## Abbreviations

BCVA: Best-corrected visual acuity
ERM: Epiretinal membrane
IOP: Intraocular pressure
logMAR: logarithm of the minimum angle of resolution
PFCL: Perfluorocarbon liquid
PPV: Pars plana vitrectomy
PVR: Proliferative vitreoretinopathy
RD: Retinal detachment

## References

[1] Scott IU, Flynn HW, Murray TG, Feuer WJ (2003). Perfluoron study group. Outcomes of surgery for retinal detachment associated with proliferative vitreoretinopathy using perfluoro-n-octane: a multicenter study. Am J Ophthalmol.

[2] Sigler EJ, Randolph JC, Calzada JI, Charles S (2013). 25-gauge pars plana vitrectomy with medium-term postoperative perfluoro-N-octane tamponade for inferior retinal detachment. OSLI Retina.

[3] Kirchhof B, Wong D, Van Meurs J, Hilgers RD, Macek M, Lois N, Schrage NF (2002). Use of perfluorohexyloctane as a long-term internal tamponade agent in complicated retinal detachment surgery. Am J Ophthalmol.

[4] Sigler EJ, Randolph JC, Calzada JI, Charles S (2013). Pars plana vitrectomy with medium-term postoperative perfluoro-N-octane for recurrent inferior retinal detachment complicated by advanced proliferative vitreoretinopathy. Retina..

[5] Mikhail MA, Mangioris G, Best RM, McGimpsey S, Chan WC (2017). Management of giant retinal tears with vitrectomy and perfluorocarbon liquid postoperatively as a short-term tamponade. Eye..

[6] Eiger-Moscovich M, Gershoni A, Axer-Siegel R, Weinberger D, Ehrlich R (2017). Short-term vitreoretinal tamponade with heavy liquid following surgery for giant retinal tear. Curr Eye Res.

[7] Zhang Z, Wei Y, Jiang X, Zhang S (2018). Surgical outcomes of 27-gauge pars plana vitrectomy with short-term postoperative tamponade of perfluorocarbon liquid for repair of giant retinal tears. Int Ophthalmol.

[8] Randolph JC, Diaz RI, Sigler EJ, Calzada JI, Charles S (2016). 25-gauge pars plana vitrectomy with medium-term postoperative perfluoro-n-octane for the repair of giant retinal tears. Graefes Arch Clin Exp Ophthalmol.

[9] Sirimaharaj M, Balachandran C, Chan WC, Hunyor AP, Chang AA, Roberts JG, Hunyo AB, Playfair TJ (2005). Vitrectomy with short term postoperative tamponade using perfluorocarbon liquid for giant retinal tears. Br J Ophthalmol.

[10] Rofail M, Lee LR (2005). Perfluoro-n-octane as a postoperative vitreoretinal tamponade in the management of giant retinal tears. Retina..

[11] Reza AT (2014). Postoperative perfluro-N-octane tamponade for complex retinal detachment surgery. Bangladesh Med Res Counc Bull.

[12] Drury B, Bourke RD (2011). Short-term intraocular tamponade with perfluorocarbon heavy liquid. Br J Ophthalmol.

[13] Rush R, Sheth S, Surka S, Ho I, Roberts JG (2012). Postoperative perfluoro-N-octane tamponade for primary retinal detachment repair. Retina.

[14] Méndez-Martínez S, Calvo P, Rodriguez-Marco NA, Faus F, Abecia E, Pablo L (2018). Blindness related to presumed retinal toxicity after using perfluorocarbon liquid during vitreoretinal surgery. Retina..

[15] Yu Q, Liu K, Su L, Xia X, Xu X (2014). Perfluorocarbon liquid: its application in vitreoretinal surgery and related ocular inflammation. Biomed Res Int.

[16] Inoue M, Iriyama A, Kadonosono K, Tamaki Y, Yanagi Y (2009). Effects of perfluorocarbon liquids and silicone oil on human retinal pigment epithelial cells and retinal ganglion cells. Retina..

